# A rare case of intra-articular synovial sarcoma of the hip joint: a case report with intra-articular findings via hip arthroscopy

**DOI:** 10.1093/jscr/rjad066

**Published:** 2023-02-27

**Authors:** Keiju Saito, Yusuke Kawabata, Naomi Kobayashi, Hiromichi Iwashita, Ikuma Kato, Masako Otani, Kenta Hayashida, Shintaro Fujita, Tomotaka Yoshida, Hyonmin Choe, Masanobu Takeyama, Yutaka Inaba

**Affiliations:** Department of Orthopaedic Surgery, Yokohama City University, Yokohama, Japan; Department of Orthopaedic Surgery, Yokohama City University, Yokohama, Japan; Department of Orthopaedic Surgery, Yokohama City University Medical Center, Yokohama, Japan; Department of Diagnostic Pathology, Yokohama City University Hospital, Yokohama, Japan; Department of Molecular Pathology, Yokohama City University Graduate School of Medicine, Yokohama, Japan; Department of Diagnostic Pathology, Yokohama City University Medical Center, Yokohama, Japan; Department of Orthopaedic Surgery, Yokohama City University, Yokohama, Japan; Department of Orthopaedic Surgery, Yokohama City University, Yokohama, Japan; Department of Orthopaedic Surgery, Yokohama City University, Yokohama, Japan; Department of Orthopaedic Surgery, Yokohama City University, Yokohama, Japan; Department of Orthopaedic Surgery, Yokohama City University, Yokohama, Japan; Department of Orthopaedic Surgery, Yokohama City University, Yokohama, Japan

**Keywords:** intra-articular sarcoma, synovial sarcoma, arthroscopy

## Abstract

Although synovial sarcoma is a relatively common soft tissue sarcoma, primary intra-articular cases are extremely rare. Herein, we report a case of primary intra-articular synovial sarcoma arising from the hip joint, that was initially treated with hip arthroscopy. A 42-year-old male presented with a history of pain in the left hip for 7 years. Radiography and magnetic resonance imaging revealed the primary intra-articular lesion and simple excision with an arthroscopy was performed. Histological findings revealed spindle cell proliferation with abundant psammoma bodies. SS18 gene rearrangement was confirmed by fluorescence *in situ* hybridization, and the tumor was diagnosed as synovial sarcoma. Adjuvant chemotherapy and radiotherapy were performed. Local control without metastasis was achieved 6 months after excision. This is the first case of intra-articular synovial sarcoma of the hip joint excised via hip arthroscopy. When an intra-articular lesion is identified, malignancies such as synovial sarcoma should be included in the differential diagnosis.

## INTRODUCTION

Synovial sarcoma is a relatively common soft tissue sarcoma representing between 2.5% and 10.5% of soft tissue sarcomas [1]. Although it tends to arise in adolescents and young adults aged 21–40 years and approximately 70% of cases occur in the extremity, especially around the knee, it can occur at any age and any location [1–4].

Nevertheless, primary intra-articular sarcomas are extremely rare [5]. Although synovial sarcoma is the most common primary intra-articular sarcoma, almost all of them occur in the knee joint [5, 6]. We here report a rare case of intra-articular synovial sarcoma in the hip joint, which was treated with the simple excision via arthroscopy.

## CASE REPORT

The patient was a 42-year-old male with a history of continuous pain in his right hip for 7 years without any kind of aggressive sports activities or trauma. He had been walking with a cane for the last year because his pain got worsened. He was referred from the initial hospital for detailed diagnosis and treatment.

**Figure 1:**
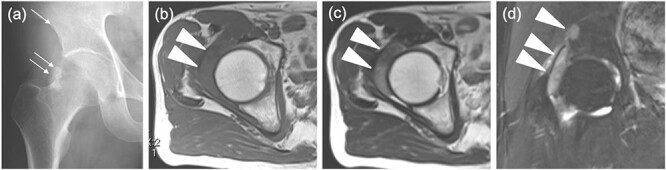
Images before the arthroscopic surgery. (**a**) A radiograph showed the calcification at the anterolateral side of the right femoral neck (two arrows) and a radiolucent shadow of the pelvic bone (an arrow). Axial (**b**) T1-, (**c**) T2-weighted image (T1WI, T2WI) and (**d**) coronal T2 fat-saturated (T2 fs) image revealed the intra-articular lesion in the right hip joint (two headless arrows). The mass was 40mm×10mm×42mm and isointense, heterogenous and hyperintense to skeletal muscle on T1WI and T2WIand T2 fs images respectively. T2WI showed triple signal intensity sign9. In addition, the coronal T2 fs image showed a pelvic bone lesion (a headless arrow).

**Figure 2:**
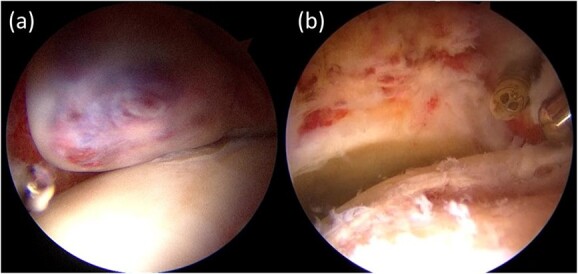
Intra-articular findings of hip arthroscopy before tumor resection (**a**) and after resection and debridement (**b**).

The radiographs showed calcification around the right femoral neck (Fig. 1a). The magnetic resonance image (MRI) showed an intra-articular lesion, which revealed that the mass was 40 ×10 × 42 mm and located at the anterolateral side of the femoral neck. The mass was isointense, slightly hyperintense and hyperintense compared with skeletal muscle on T1- and T2-weighted and T2 fat-saturated images, respectively (Fig. 1b–d). In addition, MRI showed a pelvic bone lesion, as well (Fig. 1d). At this point, we did not speculate the possibility of malignant tumor, then planned arthroscopic treatment for biopsy and tumor excision. A simple excision was performed via arthroscopic surgery, but the pelvic lesion was not excised. The arthroscopic image demonstrated a hyperemic soft tissue mass in the hip joint (Fig. 2). Removal of massive lesion and intra-articular debridement by shaver and radio frequency device was performed as much as possible.

**Figure 3:**
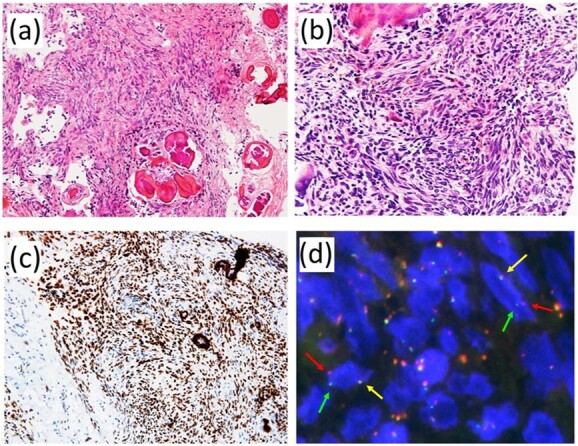
Pathological findings. (**a**) The lesion consisted of proliferation of spindle-shaped tumor cells with psammomatous calcification. (**b**) Tumor cells showed relatively monotonous appearance with plump nuclei. No mitotic figures were observed. (**c**) Immunohistochemically, tumor cells were diffusely and strongly positive for TLE1. (**d**) Fluorescence in situ hybridization using a dual-color break-apart probe covering SS18 gene exhibited one fused signal (yellow arrow) and one pair of split signals (green and red arrows).

Histologically, spindle-shaped tumor cells with plump nuclei proliferated with abundant psammomatous calcification (Fig. 3a–c). No mitotic figures were observed.

Immunohistochemically, tumor cells were diffusely and strongly positive for TLE-1 (Fig. 3c). Fluorescence *in situ* hybridization using a dual-color break-apart probe covering the *SS18* gene revealed rearrangement of the *SS18* gene (Fig. 3d). We reached a pathological diagnosis of monophasic synovial sarcoma.

**Figure 4:**
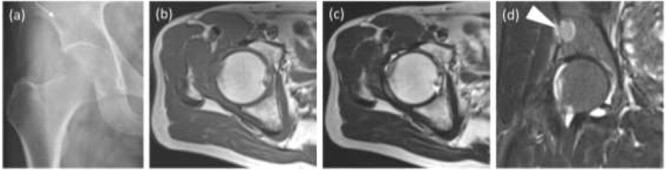
Images after the arthroscopic surgery. (**a**) The radiograph showed no calcification, which was observed before the arthroscopic surgery, and a stronger radiolucent shadow of the pelvic bone (an arrow). (**b**) T1WI axial, (**c**) T2WI axial, (**d**) coronal short T1 inversion recovery (STIR) sequences did not show the intra-articular lesion and the pelvic lesion got larger (a headless arrow).

The radiograph after the operation showed no calcification, which had been observed before the arthroscopic surgery, and showed a radiolucent shadow of the pelvic bone. The MRI showed no intra-articular lesion and that the pelvic lesion had increased in size (Fig. 4). As a postoperative treatment, the patient received adjuvant chemotherapy using doxorubicin and ifosfamide (four courses), and radiation therapy was performed after chemotherapy for local control. Thirty-five fractions of 2 Gy were administered, resulting in a total dose of 70 Gy. The radiograph after both treatments showed osteosclerosis of the pelvic lesion. Six months have passed since the end of the treatments without local recurrence or metastasis, and the patient is still undergoing follow-up and he has no pain and he can walk with no cane.

## DISCUSSION

Primary intra-articular sarcomas are extremely rare [5]. The most common primary intra-articular malignancy is synovial sarcoma [5, 6]; however, <5% of cases of synovial sarcomas originate within a joint [7]. Intra-articular sarcomas, including synovial sarcoma, arise mainly in the knee joint (approximately 30%) [5]. To our knowledge, a few reports have revealed intra-articular synovial sarcoma in the hip joint [6]. Our case is the second description of the complete clinical course.

In our case, simple excision via arthroscopy was performed, in which we firstly observed intra-articular synovial sarcoma via hip arthroscopic imaging. When intra-articular tumors are identified, it is difficult to diagnose synovial sarcoma from benign soft tissue tumors such as diffuse-type giant cell tumor (GCT) or synovial osteochondromatosis, which are more common and frequent [8].

Although specific radiological features have not been reported yet [8], radiography shows calcification in approximately 30% of synovial sarcomas, which are frequently eccentric or peripheral within the soft-tissue mass and nonspecific in appearance [9]. However, diffuse-type GCT has no calcification. Our case had calcification on the radiograph. MRI is useful in differential diagnosis; however, its capability is limited. In previous studies, synovial sarcoma was reported to have MRI characteristics frequently: (i) triple signal intensity sign (areas of hyper-, iso- and hypointensity on T2-weighted sequences), (ii) a ‘bowl-of-fruit’ appearance, (iii) fluid–fluid levels and (iv) a multilobulated appearance with intervening septa. However, other tumors also have these characteristics. Therefore, they have not been described to have a high individual specificity [6]. A 5-cm or less synovial sarcoma does not have a specific appearance [6], and our case was ~4 cm in size, had only the triple signal intensity sign and did not have the other three signs. The most important treatment for synovial sarcoma is surgical resection with negative margin. Pre- or postoperative radiation therapy has been reported to result in significant improvement in survival among synovial sarcoma patients in addition to surgical resection of synovial sarcoma [10]. While the effect of chemotherapy for synovial sarcoma is still unclear, synovial sarcoma seems to be more sensitive to chemotherapy than other sarcomas [2].

In our case, the patient underwent no wide resection but an unplanned resection with arthroscopy. After that, the patient received adjuvant chemotherapy with a first-line combination treatment: ifosfamide and doxorubicin. In addition, radiation therapy was performed after chemotherapy. MRI after chemotherapy and radiation therapy revealed no recurrence of the intra-articular lesion and that the pelvic bone lesion remained stable. In other words, chemotherapy and radiotherapy might be effective. The patient did not have local recurrence or metastatic lesions for 6 months after the end of the treatment.

## CONFLICT OF INTEREST STATEMENT

None declared.

## FUNDING

None.

## DATA AVAILABILITY

The documents used during the current study are available from the corresponding author on reasonable request.

## ETHICAL APPROVAL

All the report meets ethical guidelines and adheres to the local legal requirements.

## CONSENT STATEMENT

Written informed consent was obtained from the patient for publication of this case report and any associated images.

## GUARANTOR

K.S.
